# Functional outcome and cost-effectiveness of pulsed electromagnetic fields in the treatment of acute scaphoid fractures: a cost-utility analysis

**DOI:** 10.1186/s12891-015-0541-2

**Published:** 2015-04-11

**Authors:** Pascal F W Hannemann, Brigitte A B Essers, Judith P M Schots, Koen Dullaert, Martijn Poeze, Peter R G Brink

**Affiliations:** Department of Surgery and Traumasurgery, Maastricht University Medical Centre, PO Box 5800, 6202 AZ Maastricht, The Netherlands; Department of Clinical Epidemiology & Medical Technology Assessement (CEMTA), Maastricht University Medical Centre, PO Box 5800, 6202 AZ Maastricht, The Netherlands

**Keywords:** Scaphoid, Pulsed electromagnetic fields, Acute fractures, Cost-effectiveness analysis, Cost-utility analysis, Functional outcome

## Abstract

**Background:**

Physical forces have been widely used to stimulate bone growth in fracture repair. Addition of bone growth stimulation to the conservative treatment regime is more costly than standard health care. However, it might lead to cost-savings due to a reduction of the total amount of working days lost. This economic evaluation was performed to assess the cost-effectiveness of Pulsed Electromagnetic Fields (PEMF) compared to standard health care in the treatment of acute scaphoid fractures.

**Methods:**

An economic evaluation was carried out from a societal perspective, alongside a double-blind, randomized, placebo-controlled, multicenter trial involving five centres in the Netherlands. One hundred and two patients with a clinically and radiographically proven fracture of the scaphoid were included in the study and randomly allocated to either active bone growth stimulation or standard health care, using a placebo. All costs (medical costs and costs due to productivity loss) were measured during one year follow up. Functional outcome and general health related quality of life were assessed by the EuroQol-5D and PRWHE (patient rated wrist and hand evaluation) questionnaires. Utility scores were derived from the EuroQol-5D.

**Results:**

The average total number of working days lost was lower in the active PEMF group (9.82 days) compared to the placebo group (12.91 days) (p = 0.651). Total medical costs of the intervention group (€1594) were significantly higher compared to the standard health care (€875). The total amount of mean QALY’s (quality-adjusted life year) for the active PEMF group was 0.84 and 0.85 for the control group. The cost-effectiveness plane shows that the majority of all cost-effectiveness ratios fall into the quadrant where PEMF is not only less effective in terms of QALY’s but also more costly.

**Conclusion:**

This study demonstrates that the desired effects in terms of cost-effectiveness are not met. When comparing the effects of PEMF to standard health care in terms of QALY’s, PEMF cannot be considered a cost-effective treatment for acute fractures of the scaphoid bone.

**Trial registration:**

Netherlands Trial Register (NTR): NTR2064

## Background

The scaphoid has an essential role in proper functionality of the wrist. Fractures of the scaphoid bone are the most common fracture of the carpus, counting for up to 90% of all carpal fractures and 2% - 6% of all fractures in the Netherlands [1,2]. In Western countries the annual incidence rate of scaphoid fractures is estimated between 2.9 - 5 cases per 10.000 inhabitants [3-5]. Usually, scaphoid fractures are initially diagnosed on conventional radiographs. However, diagnosis of a scaphoid fracture can be challenging, since non- and minimally displaced fractures are often not apparent in first instance on radiographs. Timely diagnosis and accurate follow-up with the use of other diagnostic modalities, like CT-scanning, and appropriate immobilization can decrease the likelihood of occurrence of adverse outcomes like nonunion and early osteoarthritis [1,6-9]. Furthermore, delayed union leads to longer immobilization and prolonged functional deficit. Since mainly the young and working population is affected, prolonged cast immobilization leads to more working days lost with socio-economic consequences. A previous study showed that even uncomplicated healing leads to a mean total of 155 working days lost, increasing to 296 days in case of complicated healing, for instance because of nonunion [2].

Physical forces (low intensity pulsed ultrasound (LIPUS) or pulsed electromagnetic field (PEMF)) have been widely used to accelerate fracture repair in delayed union [10]. It is thought to reduce osteoclast resorption, to induce osteoid formation and to stimulate angiogenesis [11]. Evidence has accumulated over the past two decades that physical forces can also be used in the treatment of acute fractures, shortening time to union by 30% and reducing nonunion within 12 weeks of initiation of therapy by 71% [12-14].

Implementation of PEMF to the conservative treatment protocol of scaphoid fractures is more costly compared to standard care. However, it might lead to cost-savings due to a reduction of the total amount of working days lost. Therefore it is necessary to assess whether PEMF stimulation is a cost-effective treatment for acute scaphoid fractures.

This paper reports the results of our economic evaluation, performed alongside a large double-blind, randomized, placebo-controlled, multicenter trial in which the costs and effectiveness of PEMF are compared with standard care in conservatively treated acute fractures of the scaphoid bone.

## Methods

We performed an economic evaluation alongside a double-blind, randomized, placebo-controlled, multicenter trial involving five centres in the Netherlands, to establish the cost-effectiveness of pulsed electromagnetic fields (PEMF) in the treatment of acute scaphoid fractures. The clinical and radiological outcomes of the trial have been reported in detail elsewhere [15].

For the economic evaluation we used data from our randomized controlled trial and focused on functional outcome, costs and cost-effectiveness. The time horizon was similar to the clinical study from the moment of inclusion until 12 months follow-up.

Approval was obtained from the coordinating ethics review committee (Independent Review Board Nijmegen, The Netherlands) and each participating centre (Maastricht University Medical Centre, Maastricht, The Netherlands; Rijnstate Hospital, Arnhem, The Netherlands; Canisius Wilhelmina Hospital, Nijmegen, The Netherlands; Isala Clinics, Zwolle, The Netherlands; Maasziekenhuis Pantein Hospital, Boxmeer, The Netherlands). Written informed consent for participation was obtained from every patient.

### Patients

All patients > = 18 years of age diagnosed between January 1^st^ 2010 and December 31^st^ 2011 with an acute, unilateral undisplaced fracture of the scaphoid types A1, A2, B1 or B2 according to the Herbert classification [16], were included in the trial. Exclusion criteria were: displaced scaphoid fractures, proximal pole scaphoid fractures (Herbert type B3), fracture dislocations of the carpus or comminuted scaphoid fractures (Herbert type B4 and B5 fractures), presentation > five days after injury, additional fractures of the wrist, carpal or metacarpal bones, a pre-existing impairment of wrist function, pregnancy and presence of a life-supporting implanted electronic device. All scaphoid fractures were diagnosed by a combination of physical examination and radiographic imaging (conventional scaphoid series and CT scan) at the time of trauma. All included fractures were classified according to a CT-based modified Herbert classification [17]. All fractures were treated conservatively with immobilization in a below elbow cast with the first metacarpal and proximal phalanx immobilized. Electromagnetic stimulation was administered continuously for 24 hours a day using a PEMF bone growth stimulator incorporated into the cast (Orthopulse III® PEMF bone growth stimulator, Ossatec**®**, Uden, The Netherlands). The signal characteristics of the PEMF device used in our study were: pulse amplitude 50 mV, pulse width 5 μs, burst width 5 ms, burst refractory period 62 ms, repetition rate 15 Hz. Half of the PEMF-devices were randomly disabled in the factory. Identical devices and the same follow-up protocol were used for both control and PEMF cases. Neither the investigators nor the patients were aware of the device’s functionality.

The PEMF device was removed six weeks after the start of treatment. The patients underwent clinical and radiological examination (including multiplanar reconstructed CT scans) at 6, 9, 12, 24 and 52 weeks after the start of treatment. In patients with complete clinical and radiological union, the cast was removed at six, 9 or 12 weeks, depending on the time of union.

We hypothesized that PEMF would reduce the time to union by up to 30% with subsequent shortening of time off work [13]. Based on this information we conducted a power analysis with a two-sided significance level (alpha, type I error) of 0.05 and an assumed power (1-beta) of 0.8. This resulted in a total study group of 100 patients, with a sample size of 50 patients per group [18].

### Functional and quality of life outcomes

All patients were required to fill in three questionnaires at baseline, 6, 9, 12, 24 and 52 weeks after the scaphoid fracture was diagnosed. The Patient Rated Wrist/Hand Evaluation (PRWHE) was used to assess the level of fracture related functional deficit and pain level from the patient’s perspective. The PRWHE is a 15-item questionnaire that allows patients to rate the level of wrist pain and disability in activities of daily living via a 10-point categorical scale from 0 (no pain/difficulty) to 10 (worst pain ever/unable to do) [19]. The EuroQol-5-D questionnaire was used for quality of life assessment through measurement of the following six dimensions: mobility, self-care, usual activities, pain/discomfort, anxiety/depression and the visual analogue scale (VAS) [20].

### Costs

Costs were collected from the first outpatient visit until 12 months follow up and included all costs in- and outside health care. Cost analysis within health care included costs of the intervention (i.e. device, application and removal of a plaster cast), wrist/scaphoid X-rays, wrist/scaphoid CT-scans, emergency visits and outpatient clinic visits. Real resource use was collected from hospital information systems of the different hospitals. The costs per unit were derived from the financial department of our hospital and the Dutch guidelines for cost-calculations in health care [21]. Diagnostic tests (conventional radiographs and CT-scans) and outpatient clinic visits are more costly in university hospitals; therefore university and general hospital prices were used separately.

Costs concerning outside health care included productivity costs, as measured by the number of working days lost determined at baseline and 12, 24 and 52 weeks after inclusion, by the PRODISQ-questionnaire (PROductivity and DISease Questionnaire) [22]. The costs of productivity loss were calculated by means of the friction cost method, based on the average standardized wages per hour [21]. The basic idea of the friction cost method is that the amount of production lost due to disease depends on the time-span organizations need to restore the production at the initial production level [23].

No discounting was applied, since all data with regard to the costs and effectiveness were collected within one year. All costs are indexed to 2011. Table 1 provides an overview of the costs per unit.

**Table 1:** **Costs per unit** [21]

***Unit***	Cost price in academic hospital*	Cost price in general hospital*
PEMF device	665.90	665.90
Application of a cast	31.25	31.25
Removal of a cast	8.71	8.71
Wrist/scaphoid conventional radiographs	39.97	24.46
Wrist/scaphoid CT scan	126.29	80.59
Emergency department visit	154.45	154.45
Outpatient clinic visit	70.06	65.46

*Costs are indexed to 2011 and given in Euro.

### Economic evaluation

The economic evaluation was performed from a societal perspective and includes a cost-utility analysis. The incremental costs per QALY (quality-adjusted life year), usually interpreted as the additional costs of PEMF to gain one additional QALY compared to standard care, were calculated. A QALY is calculated by multiplying the utility of being in a certain health state by the time a patient experiences that health state. Utility scores were derived from the EuroQol-5D and converted to utility scores by the UK Dolan algorithm [24].

### Statistical methods

Statistical analysis was performed using proprietary statistical software with advanced statistics add-on modules (SPSS statistical package version 20.0; SPSS inc, 233S Wacker Drive, Chicago, 60606 Illinois, United States). A statistician blinded from treatment allocation was recruited for analysis of the clinical data. P-values < 0.05 were considered to indicate statistical significance. In case of normal distribution an independent student’s *t*-test was performed, to compare means between the intervention and control group. The non-parametric Mann-Whitney *U*-test was used in case of ordinal variables and in cases where normality could not be assumed.

In case of missing values, a multiple imputation model was used to analyze and complete the dataset. Missing values were below 30% for all variables. Multiple imputations for missing values concerning the questionnaires were performed using all baseline characteristics (age, sex, type of fracture, hand dominance and comorbidity) and existing variables of the EuroQol-5D and PRWHE. For each missing value, 5 imputations according to the Bayesian probability rules were performed. In addition, multiple imputations were performed for missing data on the productivity loss, again using all baseline characteristics as stated above.

Since cost data generally have a highly skewed distribution, we performed a non-parametric bootstrap analysis (1000 replications) to estimate uncertainty intervals around the difference in mean costs and to quantify the uncertainty surrounding the incremental cost-effectiveness ratio (ICER) [25,26]. The bootstrap analysis was performed in Microsoft Excel. Results of the bootstrap analysis regarding the effectiveness and costs were presented in a cost-effectiveness plane and an acceptability curve. The cost-effectiveness plane is a graphical presentation of four quadrants in which the additional costs and QALY’s of a new therapy (active PEMF) are compared to standard care. The cost-effectiveness acceptability curve shows the probability of PEMF being more cost-effective compared to standard care. All patients were analyzed according to the intention-to-treat principle.

## Results

### Patients

**Figure 1:**
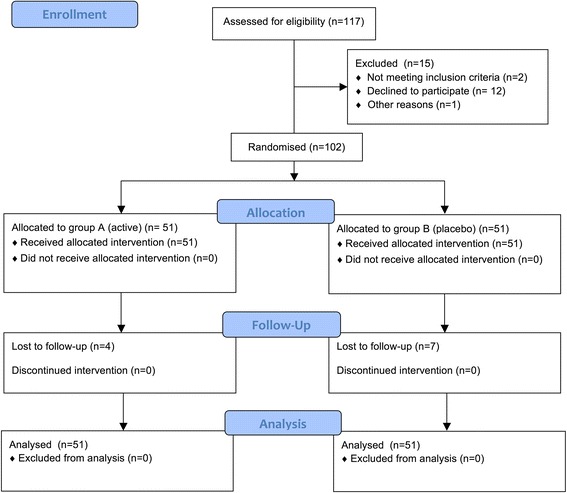
Consort flow diagram.

A total of 117 patients were eligible for inclusion; 12 patients withdrew, for two patients a primary indication for operation was set and one patient was not mentally fit enough to participate in the study. Therefore, 102 patients with clinical and radiographic diagnosis of a scaphoid fracture were included in the study and randomly allocated to either active PEMF treatment (n = 51) or placebo treatment (n = 51) (Figure 1). The two groups were similar concerning baseline characteristics (Table 2).

**Table 2:** **Baseline characteristics of the patients**

***Variable***	***Group A (Active PEMF)***	***Group B (Placebo)***
Number	51	51
Age – year*	35 (18 - 70)	34 (18 - 77)
Days between fracture and start of treatment*	4.25 (0 - 5)	3.69 (0 - 5)
Male (n, %)	40 (78)	38 (75)
Fracture in dominant hand (n,%)	28 (55)	29 (57)
Anatomical snuff box tenderness (n, %)	46 (90)	49 (96)
Pain with longitudinal compression (n, %)	39 (77)	45 (88)
Fracture type [17] (n, %)		
- Tubercle (A1)	9 (18)	9 (18)
- Transverse waist, undisplaced (A2)	19 (37)	20 (39)
- Oblique (B1)	15 (29)	18 (35)
- Transverse waist with distraction (B2)	8 (16)	4 (8)
Comorbidities (n, %)		
- None	34 (67)	38 (75)
- Osteoporosis	0 (0)	0 (0)
- Corticosteroids	0 (0)	1 (2)
- Multiple	1 (2)	1 (2)
Smoking (n, %)	16 (31)	11 (22)

*Variables are denoted as mean (range).

No significant differences were seen between groups.

### Clinical effect

Results from our recently published clinical study showed no overall positive effect of adding PEMF to the conservative treatment of acute scaphoid fractures [15]. However, the use of PEMF in A2 type scaphoid fractures revealed a significantly shorter time to union [15]. Therefore, PEMF might accelerate union in a well-defined subgroup of stable, undisplaced scaphoid waist fractures.

For the PRWHE no significant differences were found between the active PEMF group and the control group at any of the time points (Table 3). Concerning Self-Care and Anxiety/Depression, both variables of the EuroQol-5D, we found a significant difference at respectively baseline and 24 weeks. For the other variables of the EuroQol-5D there were no significant differences at any of the time points (Table 4).

**Table 3:** **Assessment of functional outcome according to the Patient-Rated Hand/Wrist Evaluation (PRWHE)**

***Variable***	***Group A (Active)***	***Group B (Placebo)***	***p-value***
Pain Subscale* (mean, [95% CI**])			
- Baseline	25.7 [21.4; 30.0]	26.8 [22.7; 31.0]	0.708
- 6 weeks	16.0 [12.4; 19.5]	15.4 [11.3; 19.4]	0.824
- 9 weeks	14.6 [11.1; 18.0]	15.4 [11.1; 19.6]	0.770
- 12 weeks	10.7 [7.4; 14.0]	10.2 [6.6; 13.9]	0.851
- 24 weeks	8.3 [4.9; 11.7]	7.2 [4.3; 10.1]	0.631
- 52 weeks	4.6 [2.1; 7.1]	3.7 [1.1; 6.3]	0.626
Function Subscale^†^ (mean, [95% CI**])			
- Baseline	74.3 [67.9; 81.0]	68.1 [59.8; 76.3]	0.235
- 6 weeks	52.2 [44.3; 60.1]	47.5 [38.4; 56.5]	0.426
- 9 weeks	29.3 [21.0; 37.6]	34.9 [23.5; 46.3]	0.411
- 12 weeks	16.6 [9.3; 23.9]	20.8 [12.2; 29.3]	0.456
- 24 weeks	8.1 [4.0; 12.1]	6.4 [2.1; 10.7]	0.569
- 52 weeks	2.8 [0.8; 4.7]	3.4 [0.5; 6.2]	0.724
Total Score^‡^ (mean, [95% CI**])			
- Baseline	62.9 [56.5; 69.3]	60.9 [53.1; 68.7]	0.687
- 6 weeks	42.1 [35.5; 48.7]	39.1 [31.0; 47.2]	0.565
- 9 weeks	29.2 [22.1; 36.4]	32.8 [23.4; 42.2]	0.531
- 12 weeks	19.0 [12.6; 25.4]	20.6 [13.2; 28.0]	0.738
- 24 weeks	12.3 [7.1; 17.6]	10.4 [5.7; 15.1]	0.586
- 52 weeks	5.9 [2.7; 9.2]	5.4 [1.5; 9.3]	0.832

*Best possible outcome (no pain) = 0, worst possible outcome = 50.

**CI, confidence interval.

^†^Best possible outcome (no functional disability) = 0, worst possible outcome = 100.

‡Best possible outcome (no pain & no functional disability) = 0, worst possible outcome = 100.

**Table 4:** **Assessment of functional outcome according to the EuroQoL-5D**

***Variable***	***Group A (Active)***	***Group B (Placebo)***	***p-value***
Mobility* (mean rank**)			
- Baseline	49.35	43.52	0.052
- 6 weeks	43.28	39.54	0.115
- 9 weeks	39.19	38.76	0.848
- 12 weeks	38.00	37.00	0.646
- 24 weeks	37.54	36.54	0.597
- 52 weeks	36.34	34.50	0.191
Self-Care* (mean rank**)			
- Baseline	51.34	41.44	**0.039**
- 6 weeks	44.51	37.22	0.111
- 9 weeks	37.24	41.22	0.340
- 12 weeks	37.42	37.58	0.960
- 24 weeks	37.00	37.00	1.00
- 52 weeks	35.29	35.00	0.359
Usual activities* (mean rank**)			
- Baseline	48.6	44.3	0.373
- 6 weeks	42.3	40.6	0.704
- 9 weeks	38.9	39.2	0.945
- 12 weeks	35.9	39.1	0.441
- 24 weeks	38.4	35.5	0.309
- 52 weeks	37.0	33.7	0.215
Pain/discomfort* (mean rank**)			
- Baseline	49.3	43.6	0.250
- 6 weeks	44.3	38.4	0.195
- 9 weeks	39.1	37.8	0.769
- 12 weeks	39.4	35.7	0.379
- 24 weeks	40.7	33.2	0.051
- 52 weeks	37.3	33.4	0.234
Anxiety/depression* (mean rank**)			
- Baseline	47.8	45.1	0.339
- 6 weeks	40.9	42.2	0.625
- 9 weeks	37.8	40.5	0.251
- 12 weeks	35.5	39.5	0.091
- 24 weeks	35.0	39.1	**0.038**
- 52 weeks	34.4	36.8	0.229
VAS-score^†^ (mean, [95% CI^‡^])			
- Baseline	72.5 [65.9; 79.0]	78.7 [73.9; 83.4]	0.126
- 6 weeks	78.0 [73.8; 82.3]	82.6 [78.4; 86.8]	0.127
- 9 weeks	82.2 [78.3; 86.1]	82.2 [77.2; 87.3]	0.987
- 12 weeks	84.8 [80.7; 89.0]	82.9 [77.6; 88.3]	0.576
- 24 weeks	88.1 [83.9; 92.2]	88.7 [85.0; 92.4]	0.818
- 52 weeks	87.9 [84.1; 91.8]	90.8 [87.0; 94.7]	0.282

*Mann Whitney *U* test; p < 0.05.

**Scores are presented as mean rank. The higher the score, the more problems experienced with activities of daily living.

^†^All variables are denoted as a score between 0-100, with 100 being the best possible outcome.

^‡^CI, confidence interval.

### Costs

The mean total health care costs per patient were €875 in the placebo group and €1594 in the active PEMF group (Table 5). This difference in costs was statistically significant (€719, 95% CI €652 to €772) (Table 6) and could be attributed entirely to the use of the PEMF device.

**Table 5:** **Resource use and costs per treatment arm**

***Unit***	***Group A (active PEMF)***		***Group B (Placebo)***	
	**Mean total use (n)**	Mean total costs*	**Mean total use (n)**	Mean total costs*
PEMF device	1.0	665.90	0	0
Application of a cast	2.61	81.50	2.53	79.04
Removal of a cast	2.61	22.71	2.53	22.03
Wrist / scaphoid conventional radiographs	1.0	30.85	1.0	31.15
Wrist / scaphoid CT scan	2.95	276.78	2.65	255.35
Emergency department visit	1.0	154.45	1.0	154.45
Outpatient clinic visit	5.12	345.46	4.69	317.23
Total health care costs**		€1594		€875

*Costs are indexed to 2011 and denoted in Euro.

**Total health care costs do not exactly add up due to the bootstrap analysis.

**Table 6:** **Total costs per treatment arm**

	***Group A (Active PEMF) mean***	***Group B (Placebo) mean***	***Difference in costs [95% CI]***
Total health care costs*	€1594	€875	€719 [652; 772]
Costs of productivity los	€1226	€1423	€-197 [-1158; 657]
Total societal costs***	€2827	€2253	€574 [-423; 1438]

*Costs are denoted in Euro.

**CI, confidence interval.

***Numbers do not exactly add up due to the bootstrap analysis.

Concerning productivity loss the average total number of working days lost was lower in the active PEMF group (9.82 days) compared to the placebo group (12.91 days). However, this difference was not statistically significant (p = 0.651). When including costs due to productivity loss in the cost-analysis, mean total costs for the active PEMF group are higher (€2827) compared to mean total costs for the standard treatment (€2253), although this difference is not statistically significant (difference = €574, 95% CI = €-423 to €1438) (Table 6).

When excluding patients from the analysis that were student or unemployed (17 in the active PEMF group and 12 in the placebo group, p = 0.27), mean total costs for the active PEMF group are still higher (€3305) compared to mean total costs for the standard treatment (€2634) (difference = €670, 95% CI = €-405 to €1732) (Table 7).

**Table 7:** **Total costs per treatment arm for working patients only**

	***Group A (Active PEMF) mean***	***Group B (Placebo) mean***	***Difference in costs [95% CI]***
Total health care costs*	€1543	€840	€703 [637; 755]
Costs of productivity los	€1747	€1798	€-51 [-1119; 975]
Total societal costs***	€3305	€2634	€670 [-405; 1732]

*Costs are denoted in Euro.

**CI, confidence interval.

***Numbers do not exactly add up due to the bootstrap analysis.

### Cost-effectiveness

**Figure 2:**
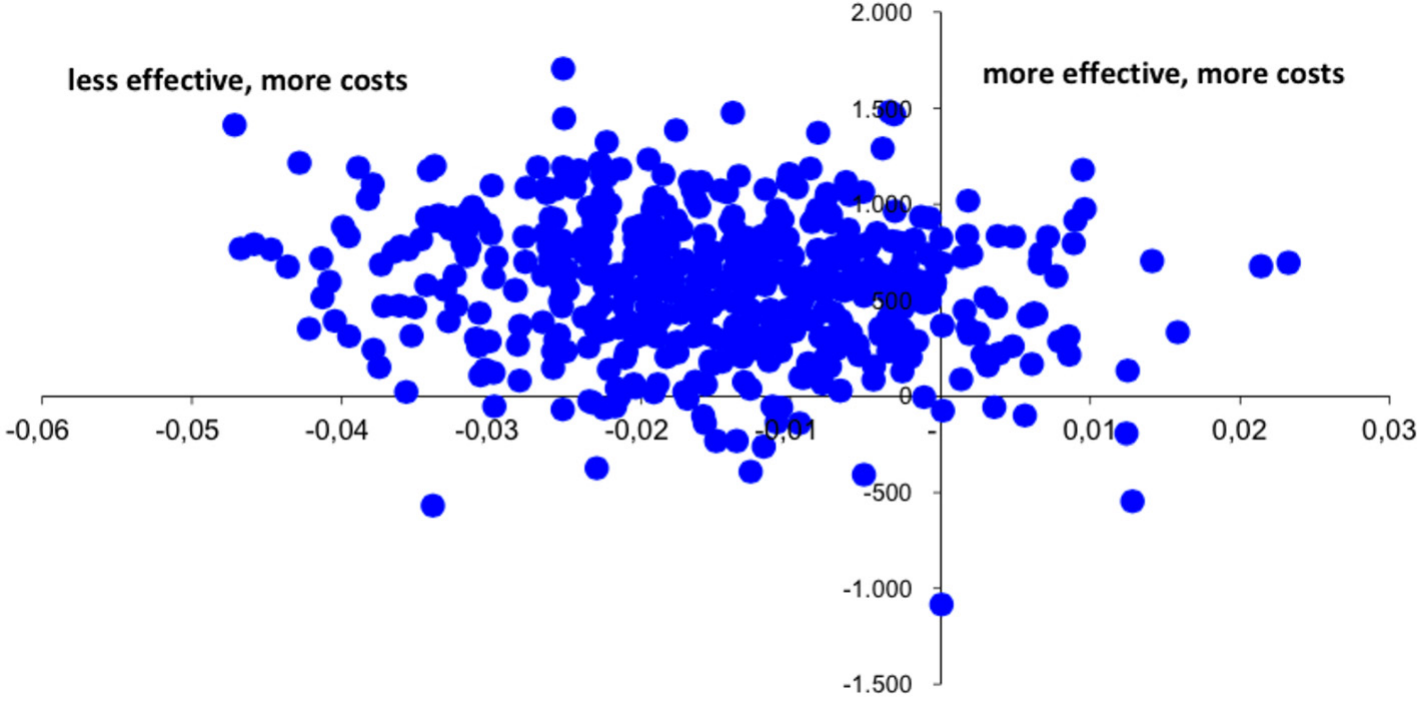
Cost-effectiveness plane for the incremental costs per QALY (based on EuroQol-5D).

**Figure 3:**
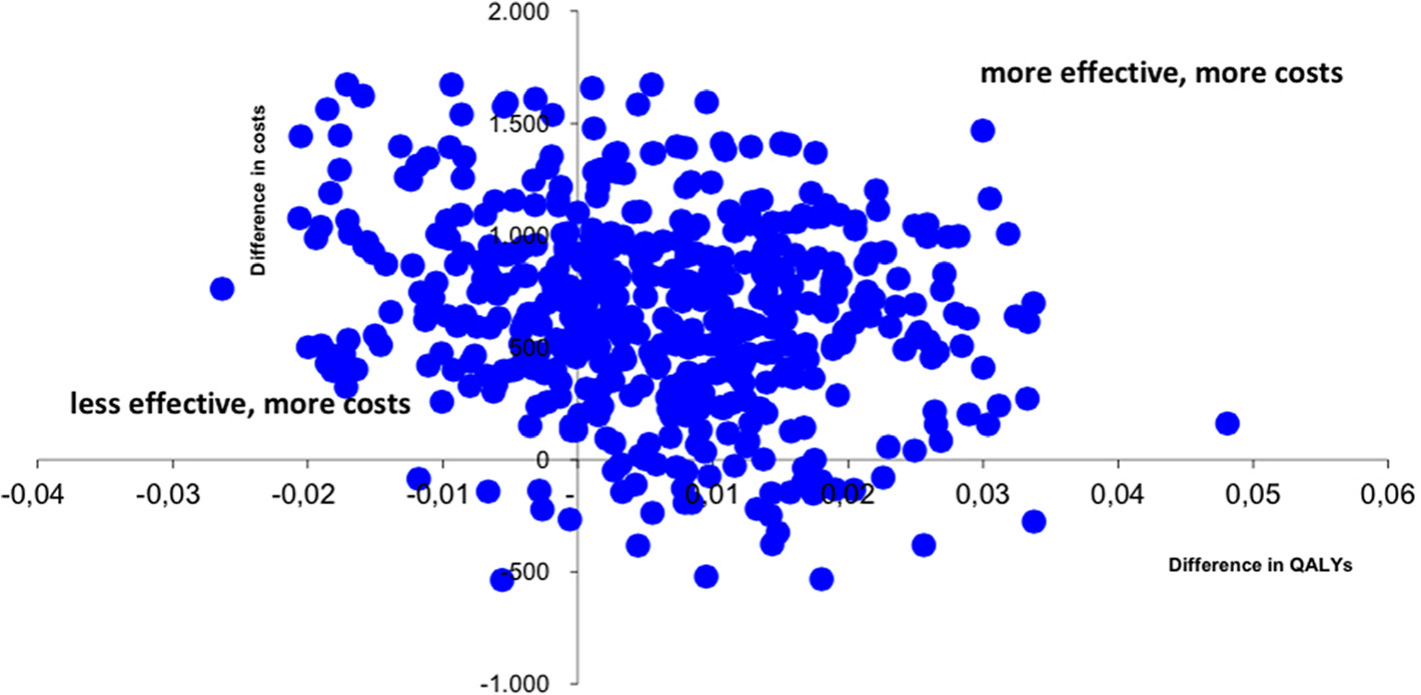
Cost-effectiveness plane for the incremental costs per QALY (based on EuroQol-5D) for working patients only.

**Figure 4:**
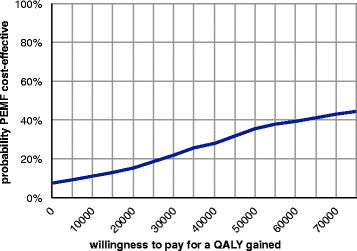
Cost-effectieveness acceptability curve (based on EuroQol-5D) for working patients only.

The mean QALY was 0.84 for the active PEMF group and 0.85 for the control group, resulting in a non-significant difference of 0.01 (95% CI = -0.01;0.04) (Table 8). The cost-effectiveness plane (Figure 2) shows that the majority of all cost-effectiveness ratios (85%) fall into the north-western quadrant where PEMF is less effective in terms of QALY’s and more costly. When only considering patients with a paid job, the difference in QALY’s was in favour of the PEMF group (non-significant difference: 0.01: 95% CI = -0.04;0.02) which results in an incremental cost-effectiveness ratio of €113559 per QALY gained which is unlikely to be considered cost-effective (Table 9). In addition, the cost-effectiveness plane shows that although 64% of the cost-effectiveness pairs are located in the north-eastern quadrant where active PEMF treatment leads to more effect against more costs, 29% is situated in the quadrant where PEMF is less effective and more costly (Figure 3) The acceptability curve (Figure 4) shows that even for a threshold up to €70000, there is only a 40% probability that PEMF is more cost-effective compared to standard care.

**Table 8:** **Incremental cost-effectiveness ratio of PEMF vs. standard treatment for acute scaphoid fractures**

	Costs*	***QALY***	***ICER***
Group A (Active)	€2827	0.84	
Group B (Placebo)	€2253	0.8545	
Increment	€574	-0.0145	inferior

*Costs are denoted in Euro.

QALY, quality adjusted life year; ICER, incremental cost-effectiveness ratio.

**Table 9:** **Incremental cost-effectiveness ratio of PEMF vs. standard treatment for acute scaphoid fractures for working patients only**

	Costs*	***QALY***	***ICER***
Group A (Active)	€3305	0.8474	
Group B (Placebo)	€2634	0.8415	
Increment	€670	0.0059	113559

*Costs are denoted in Euro.

QALY, quality adjusted life year; ICER, incremental cost-effectiveness ratio.

## Discussion

Data about the amount of working days lost due to scaphoid fractures is limited. The most detailed source is a previously published prospective study that assessed, among other things, time off work due to carpal injuries in the Netherlands in the period from 1990 to 1993 [2]. In this study the mean total time off work for conservatively treated fractures of the scaphoid was 144 days with a range of 12 to 1353 days. In our trial the mean total number of working days lost was remarkably lower, 9.82 working days in the active PEMF group compared to 12.91 working days lost in the control group with a range of 0 to 190 days.

In the study by van der Molen et al. [2] the majority of the included patients (94%) performed manual work whereby they needed both hands for their job. Only 2% of the study population was able to resume their previous job with an immobilized wrist. All 533 patients were off work for at least one day.

In contrast, in our study 55% of the included patients performed manual work and 49% of the responders with a paid job resumed their jobs immediately with an immobilized wrist and therefore had no working days lost. This suggests that the most important factor influencing time off work among patients with immobilized wrists due to carpal injuries is the patient’s occupation. In accordance with other authors, we concluded that non-manual workers had less time off work than manual workers [27].

The average total number of working days lost was lower in the active PEMF group compared to the placebo group. However, we could only retrieve information concerning the number of working days lost in 73% of the study population. In total, 49 out of 74 patients had no working days lost; 26 out of 39 patients in the active group compared to 23 out of 35 patients in the control group. Since 49% of the responders with a paid job was able to return to their previous job immediately, and only 11% was off work for more than one month, the costs due to productivity loss were less than we originally expected on basis of previously best available evidence from van der Molen et al. [2]. Moreover, this study was based on data from 1991 to 1993. Properties and demands regarding manual work have changed over this period of almost 20 years.

A number of patients were student or unemployed and therefore not generating a loss in working days in the context of a paid job. Although the difference in unemployed patients between groups was not statistically different (p = 0.27), we also calculated costs due to productivity loss and total costs among working patients only. Obviously, the number of working days lost was higher amongst working patients (15.32 days in the PEMF group vs. 18.83 days in the placebo group, p = 0.73) resulting in higher costs due to loss of productivity. However, mean total costs were still higher in the PEMF group. Combining the costs and effects for the subgroup of working patients resulted in an incremental cost-effectiveness ratio of €113559 per QALY gained which is unlikely to be considered cost-effective given the commonly applied Dutch threshold value of €40000 [28].

Our research group performed the first CT-scan evaluated, double-blind, randomized, placebo-controlled trial to investigate the effect of PEMF bone growth stimulation on the time to union, functional outcome and cost-effectiveness of acute fractures of the scaphoid. We found no conclusive evidence supporting the implementation of PEMF stimulation to the conservative treatment regime [15,29]. As reported in our recently published study concerning the clinical section of the study, the addition of PEMF stimulation to the conservative treatment of acute scaphoid fractures might accelerate union in a well-defined subgroup of stable undisplaced scaphoid waist fractures [15]. However, it does not improve overall functional and radiological outcomes of conservatively treated scaphoid fractures. These results are not consistent with the results of former acute fracture studies on the use of electrical bone growth stimulation. Heckman et al. reported in a randomized controlled trial concerning addition of LIPUS to the treatment protocol of acute diaphyseal tibia fractures, a significant decrease in time to union (p = 0.0001) and substantial cost-savings [12]. Kristiansen et al. compared LIPUS with standard care in non-operatively treated extra-articular distal radius fractures. The time to union was accelerated by 37 days (p < 0.0001) and LIPUS treatment was associated with a significantly smaller loss of reduction (p < 0.01) [14]. Mayr et al. reported on acute nondisplaced scaphoid fractures (Herbert B1 and B2) treated with LIPUS. Results showed a significant acceleration of the fracture-healing process by 17 days (p < 0.01). Furthermore, there was significantly more trabecular bridging after six weeks in the LIPUS group (81,2%) compared to the placebo group (54,6%) [13].

A possible explanation for the difference in clinical outcome compared to previous literature and a limitation of our study may be the large heterogeneity of our study group, since we included different types of scaphoid fractures (Herbert types A1, A2, B1 and B2). Post-hoc log-rank analysis of all data from our recently published clinical study revealed a significantly shorter time to union in the active PEMF group for undisplaced transverse scaphoid waist fractures (Herbert type A2 fractures) [15,16]. PEMF bone growth stimulation seems to have an accelerating effect on union in stable scaphoid waist fractures. Since unstable fractures are more likely to progress to nonunion, we think that stability of the fracture pattern may play a crucial role in predicting the effect of PEMF bone growth stimulation in scaphoid waist fractures. Therefore, electrical bone growth stimulation might be only effective in a well-defined subgroup of stable scaphoid fractures. Future studies should focus on this subgroup of scaphoid fractures to evaluate whether the use of PEMF is clinically effective and therefore justified among patients with this type of fracture. This seems particularly relevant since recent research has shown that the majority of scaphoid waist fractures are undisplaced [30].

Concerning the functional outcome as measured by the PRWHE questionnaire and general health related quality of life, as measured by the EuroQol-5D, we found no significant differences between the active PEMF group and the control group. Our cost-effectiveness analysis shows that PEMF stimulation significantly increases treatment costs against no significant improvement of general health related quality of life.

## Conclusions

In conclusion, evidence from our clinical trial has not demonstrated sufficient advantage to warrant routine use of PEMF, although PEMF might accelerate union in a well-defined subgroup of stable, undisplaced scaphoid waist fractures. From a societal perspective, we conclude that the desired effects in terms of cost-effectiveness are not met. When comparing the effects of PEMF to placebo expressed in QALY’s, PEMF cannot be considered a cost-effective treatment for acute fractures of the scaphoid bone in general.

## Abbreviations

PEMF: Pulsed electromagnetic fields
PRWHE: Patient rated wrist and hand evaluation
QALY: Quality adjusted life year
LIPUS: Low intensity pulsed ultrasound
VAS: Visual analogue scale
PRODISQ: PROductivity and DISease Questionnaire
